# Correlated evolution of leaf and root anatomic traits in *Dendrobium* (Orchidaceae)

**DOI:** 10.1093/aobpla/plaa034

**Published:** 2020-07-20

**Authors:** Ying Qi, Jia-Lin Huang, Shi-Bao Zhang

**Affiliations:** 1 Key Laboratory for Economic Plants and Biotechnology, Kunming Institute of Botany, Chinese Academy of Sciences, Kunming, Yunnan, China; 2 University of Chinese Academy of Sciences, Beijing, China

**Keywords:** Co-evolution, elevation, epiphyte, water conservation, water shortage

## Abstract

The whole-plant economic spectrum concept predicts that leaf and root traits evolve in coordination to cope with environmental stresses. However, this hypothesis is difficult to test in many species because their leaves and roots are exposed to different environments, above- and below-ground. In epiphytes, both leaves and roots are exposed to the atmosphere. Thus, we suspect there are consistent water conservation strategies in leaf and root traits of epiphytes due to similar selection pressures. Here, we measured the functional traits of 21 species in the genus *Dendrobium*, which is one of the largest epiphytic taxa in the family Orchidaceae, and used phylogenetically independent contrasts to test the relationships among traits, and between traits and the environment. Our results demonstrate that species with a thicker velamen tended to have thicker roots, a thicker root cortex and vascular cylinder, and a larger number of vessels in the root. Correspondingly, these species also had higher leaf mass per area, and thicker leaf lower cuticles. Leaf and root traits associated with water conservation showed significantly positive relationships. The number of velamen layers, leaf density and the ratio of vascular cylinder radius to root radius were significantly affected by the species’ differing environments. Thus, traits related to water conservation and transport may play an important role in helping *Dendrobium* cope with the cool and dry conditions found at high elevations. These findings confirmed the hypothesis that leaf and root traits have evolved in coordination, and also provide insights into trait evolution and ecological adaptation in epiphytic orchids.

## Teaser

Our study proposed a model of interaction between leaf and root traits of *Dendrobium* which is an important epiphytic taxon in Orchidaceae. Both leaves and roots of epiphytes are exposed to the atmosphere; thus, these two organs are under similar selective pressures. To cope with water stress in epiphytic habitats, leaf and root traits associated with water conservation show significantly positive relationships in *Dendrobium* species, and have evolved in coordination. Our findings provide insights into trait evolution and ecological adaptation in epiphytic orchids.

## Introduction

Trade-offs among functional traits reveal the strategies for plants to acquire and conserve resources (Wright and Westoby 2002; Kong *et al.* 2015), and provide insights into species distribution and ecosystem processes (Fortunel *et al.* 2012). These functional traits have been described as the ‘spectra’ to separate species with different adaptation strategies (Liese *et al.* 2017). On one end of the ecological axis are species with an acquisitive strategy. These species with low leaf mass per area (LMA) have higher photosynthetic rates but shorter lifespans (Reich *et al.* 1998; Westoby *et al.* 2002). On the other end of this axis are species with a conservative strategy. These species with denser tissue have greater resistance to mechanical damage and pathogen attack, leading to slower growth rates and longer lifespans (Poorter *et al.* 2008; Liu *et al.* 2010; Kong *et al.* 2015). Key traits related to resource acquisition and conservation should be considered as a part of the leaf and root functional coordination (Freschet *et al.* 2015).

The leaf economic spectrum (LES) concept has been widely applied. This concept hypothesizes that leaf functional traits may co-vary along a distinct spectrum among species (Wright *et al.* 2004; Somavilla *et al.* 2013; Poorter *et al.* 2014). Within the literatures on LES, similar trait spectra have been expanded to stems and roots, thus forming the whole-plant economic spectrum (Freschet *et al.* 2010; Kong *et al.* 2015; Díaz *et al.* 2016; Valverde-Barrantes *et al.* 2017). Although the root is the main organ for resource acquisition, root traits receive the least attention in plant ecology research (Manschadi *et al.* 2006; Liese *et al.* 2017; Valverde-Barrantes *et al.* 2017; Kong *et al.* 2019). Research on root traits has been hampered due to constraints in observation and sampling, such that plant roots are labelled ‘the hidden half’ (Eshel and Beeckman 2013). Another reason for the complexity in evaluating the root trait syndrome is the linkage between leaf and root traits (Withington *et al.* 2006; Mommer and Weemstra 2012). According to the whole-plant economic spectrum hypothesis, leaf and root traits evolve in coordination (Freschet *et al.* 2010). However, studies on relationships between leaf and root traits across species have showed contrasting results. For example, Craine and Lee (2003) found nitrogen concentration and tissue density of leaves are correlated with those of fine roots. Tjoelker *et al.* (2005) found a concordance in leaf and root longevity. However, Withington *et al.* (2006) suggested tissue structure and longevity above-ground (leaves) can contrast markedly with those of below-ground (roots). Thus, more research into root traits is needed to resolve these contrasting findings. The decoupling of leaf and root traits may be caused by the following reasons. Firstly, differences in plant growth form may affect trait correlations (Reich *et al.* 1998; Withington *et al.* 2006; Liu *et al.* 2010). For example, among grass species, the acquisitive strategy is associated with low LMA, low leaf tissue density and low root tissue density (Ryser *et al.* 1997; Wahl and Ryser 2000), whereas among tree species, acquisitive strategy is associated with higher specific root length and smaller root diameters, but not root tissue density (Comas *et al.* 2002). This suggests that the trait correlations or plant strategies that have been widely observed in herbaceous plants cannot be directly extrapolated to woody plants (Liu *et al.* 2010). Secondly, the drivers of morphological variation in leaf and root traits may be different (Kembel and Cahill 2011; Valverde-Barrantes *et al.* 2017). Previous studies have suggested that phylogeny plays a major role in root trait variation (Kong *et al.* 2014; Reich 2014), whereas environmental factors may largely account for variations in leaf traits (Baraloto *et al.* 2012). Thus, when examining species-level responses to environmental changes, phylogeny should be considered (Ackerly and Donoghue 1998; Edwards 2006). Furthermore, leaf and root traits may be decoupled due to the differences in above- and below-ground environments (Freschet *et al.* 2015; Adair *et al.* 2019). For example, the availabilities of nutrients and water in soil are significantly higher, and more stable than that in atmosphere or canopy (Zotz *et al.* 2010). However, it is not clear whether the association between leaf and root traits of epiphytes is stronger than that of terrestrial plants.

The roots of tree- and rock-dwelling epiphytes are exposed to similar environments as their leaves (Zotz and Winkler 2013). Epiphyte habitats supply irregular amounts of water, and the resultant water stress strongly inhibits epiphyte growth and survival (Zotz 2005; Zotz *et al.* 2010). In response to frequent drought stress, epiphytes have evolved ecophysiological adaptations (Zhang *et al.* 2018). Specifically, the aerial roots of epiphytes capture water via a special spongy structure called velamen, which absorbs water that flows down the tree trunk or rock surface (Roberts and Dixon 2008). Although velamen is not exclusive to epiphytes (Zotz *et al.* 2017), its role in epiphytes’ physiology is especially important. Thick velamen significantly delays water loss (Zotz and Winkler 2013), allowing epiphytes to survive in habitats where few other plants can survive, such as habitats with extremely small amounts of water availability (Roberts and Dixon 2008; Zotz and Winkler 2013; Joca *et al.* 2017). Plants can also respond to water availability by adjusting leaf traits (Wright *et al.* 2005; Qin *et al.* 2019). For example, plants can adapt to water shortage by regulating their stomatal area (SA), stomatal density (SD), leaf density (LD) and epidermis or cuticle thickness (Zhang *et al.* 2012). Although velamen has an important role in water conservation, few researches have tested the coordination between velamen thickness (VT) and leaf traits related to water conservation (Zotz and Winkler 2013). Thus, it would be valuable to explore whether both leaf and root traits follow accordant trends in their water conservative strategies.

To address whether leaf and root traits in epiphytes show coordinated evolution in response to changing environments, we analysed the variations in leaf and root traits in species of the genus *Dendrobium*. All members within the genus are epiphytic or lithophytic (Zhu *et al.* 2009), and have roots that are easily observed and sampled. In addition, *Dendrobium* is one of the largest genera in Orchidaceae, and presents some of the most intricate taxonomic problems in the family (Xiang *et al.* 2013). Whether *Dendrobium* is monophyletic have been inconclusive to date (Schuiteman 2011; Takamiya *et al.* 2014). The phylogenetically independent contrast (PIC) method has been widely used in ecology to detect the evolutionary correlation among traits (Price 1997), because ignoring phylogenetic relationships among species included in a comparative analysis may lead to spurious conclusions due to high type I or type II errors (Morand and Poulin 2003). The correlated evolution between traits has been tested in large taxa by using a PIC method (e.g. angiosperm) or specific clades (Grotkopp and Rejmánek 2007; Fortunel *et al.* 2012; Zhang *et al.* 2012). However, previous studies into relationships among traits, and between plant traits and environmental factors in epiphytes mostly focused on above-ground organs, with particular emphasis on leaf traits, but rarely on the roots (Sun *et al.* 2014; Teixeira da Silva *et al.* 2016). The leaves and roots of epiphytes may experience similar selection pressures, but no study has been conducted to detect the evolutionary association between leaf and root traits of epiphytes, including *Dendrobium*.

Here, we determined the patterns of variation for 36 leaf and root traits in 21 species of *Dendrobium*, and used the PIC method to detect whether species traits co-varied with other traits and/or with the environment, and tried to answer following questions: (i) How do leaf and root traits vary with velamen thickness? (ii) Are there close associations between leaf and root traits in *Dendrobium* species? (iii) Are leaf and root traits shaped by phylogeny? We suspect leaf and root traits related to water conservation will coordinate along single axes of resource acquisition/conservation in *Dendrobium* species when their leaves and roots are exposed to similar environments.

## Materials and Methods

### Plant materials and study site

Twenty-one (21) species of *Dendrobium*, including epiphytes and lithophytes, were cultivated in a greenhouse at the Kunming Institute of Botany, Chinese Academy of Sciences (elevation 1990 m, 102°41′E, 25°01′N). Two species, *D. kingianum* and *D. bracteosum*, were collected from Australia. Nine species (*D. loddigesii*, *D. nobile*, *D. longicornu*, *D. crystallinum*, *D. crepidatum*, *D. chrysanthum*, *D. fimbriatum*, *D. chrysotoxum* and *D. thyrsiflorum*) were collected from the Xishuangbanna Tropical Botanical Garden, Chinese Academy of Sciences, and the remaining 11 species were grown at the Kunming Institute of Botany. Information on the natural habitat, growth form and altitude of the species was sourced from the Flora of China (Zhu *et al.* 2009; http://www.efloras.org), Teixeira da Silva *et al.* (2016) and Simpson *et al.* (2018). To ensure that interspecific differences were not merely the result of plastic responses to variable growth conditions, plants were grown for >1 year in a greenhouse at the Kunming Institute of Botany. Plants were grown on a substrate that consisted of a mixture of 70 % bark (1 cm × 1 cm), 20 % moss and 10 % humus, at 18–27 °C, with a relative humidity of 50–70 %, and 20 % full sunlight. Water and fertilizer were supplied as needed. To avoid changes in root structure due to substrates, only aerial roots were selected as our test material.

### Phylogenetic tree

A Phylogram was generated using concatenated data sets of nucleus gene: Internal Transcribed Spacers (ITS) and the chloroplast genes: *rbc*L, *mat*K–*trn*K, *trn*H–*psb*A regions which were downloaded from GenBank (http://www.ncbi.nlm.nih.gov). *Bulbophyllum odoratissimum* was chosen as the outgroup because of its close relationship to *Dendrobium* (Freudenstein and Rasmussen 1999; Xiang *et al.* 2013). Numbers associated with nodes are maximum-likelihood bootstrap values. Multiple alignments were automatically performed using ClustalX v.2.0.11 and manual corrections through BioEdit v.7.0.9.0, generating a matrix in a NEXUS format for Bayesian analyses in MrBayes v3.2.2 x64. These analyses used the best-fit models selected with model selection criterion AIC by the software jModeltest v.2.1.4. In the Bayesian analyses, trees were generated by running Metropolis-coupled Monte Carlo Markov (MCMC) chains and sampling one tree every 100 generations for 1 000 000 generations, starting with a random tree. The phylogenetic relationships of the studied *Dendrobium* species and their ecological information are shown in **Supporting Information—Fig. S1**.

### Sampling and measurement

To minimize the confounding effect of plant age, for each species, at least six mature individuals were randomly selected, and three healthy, mature leaves and roots from each individual were collected. Leaves were selected in the middle part of the leaf (avoiding the main vein) and roots were sampled ~2 cm above the apex of new viable roots. After collection, samples were sealed in plastic bags, and anatomical traits were immediately measured. Collection and measurement were conducted during the wet season (from July to September 2018).

After measuring the fresh mass (*M*_L(F)_) of leaves, the leaf area (LA) was measured with a Li-Cor 3000A area meter (Li-Cor Inc., Lincoln, NE, USA), and leaves were then oven-dried for 48 h at 70 °C until reaching a constant mass to obtain leaf dry mass (*M*_L(D)_). Water content (WC, %) was calculated as (*M*_L(F)_ − *M*_L(D)_)/*M*_L(F)_ × 100 %. Leaf mass per area (LMA) was calculated as *M*_L(D)_/LA.

To characterize leaf anatomical traits, we cut 5-mm × 2-mm sections from the middle part of the leaf (avoiding the main vein) with a freezing microtome (CM3050S, Leica, Germany). The sections were observed and photographed under an optical microscope (DM2500, Leica, Germany). Leaf thickness (LT), upper epidermal thickness (UET), lower epidermal thickness (LET), upper cuticle thickness (UCT) and lower cuticle thickness (LCT) were measured with the software ImageJ v.1.43u (National Institutes of Health, Bethesda, MD, USA). Leaf density (LD, kg m^−3^) was calculated as leaf dry mass per unit volume, which was calculated as LA × LT (Sun *et al.* 2014).

 For stomatal traits, abaxial nail varnish peels were taken centrally, midway between the midrib and margin (Sack *et al.* 2003), transferred to glass slides after drying and then photographed under an optical microscope. The images were measured using ImageJ. Stomatal density (SD) was measured as the number of stomata per unit area, and was calculated as the mean value of >36 images from each species (6 images per leaf). Stomatal length (SL) and width (SW) were averaged from 60 randomly selected stomata for each species. Stomatal area (SA) was estimated by the formula 1/4 × π × SL × SW (Sun *et al.* 2014).

To measure vein density (VD), the leaves were boiled for 30 min in 5 % NaOH and washed with distilled water three times, then bleached in 5 % sodium hypochlorite until the mesophyll was transparent. The leaves were then stained for 2 min with 1 % toluidine blue, mounted on glass slides and photographed. Total vein length was measured with ImageJ, and VD was calculated as total vein length per leaf area (LA).

To examine root anatomical traits, we used a freezing microtome to cut 4-mm-thick sections ~2 cm from the root apex and photographed the cross sections with an optical microscope. Velamen thickness (VT) and root radius (*r*) were measured with ImageJ. The area of velamen (*A*_vel_) was calculated as the whole cross-section area minus the area within the epidermis. We measured the length (vcl) and width (vcw) of ~100 randomly selected velamen cells. The area of each velamen cell (*A*_vc_) was calculated as vcl × vcw. Exodermic, endodermic and passage cells were counted using ImageJ. The number of vessel (*N*_ves_) refers to the number of primary xylem vessels. To determine vessel diameter (*D*_ves_) and vessel area (*A*_ves_), we measured all the primary xylem vessels.

### Data analysis

Before analysis, all data were log10 transformed to improve normality and homoscedasticity. Comparison of traits among different groups was conducted by a one-way ANOVA. A PIC method was used to detect whether species traits co-varied with other traits or with the environment (Price 1997; Purvis and Webster 1999) by employing the ‘ape’ package in R v.3.4.4. Any PIC correlations were evaluated with a ‘Pearson’ correlation in R package.

To evaluate the evolutionary history of leaf and root traits, we first tested for a phylogenetic signal in each trait using the *K*-statistic, which is based on a ‘Brownian motion model’ of trait evolution (Blomberg *et al.* 2003). The *K* metric can be used to assess phylogenetic conservatism. *K* > 1 indicates that a trait value is more conserved than expected from Brownian motion. *K* < 1 indicates that a trait value is significantly less conserved than expected from Brownian motion, and instead demonstrates significant lability, while *K* = 1 shows that a trait value is as expected from a Brownian motion model (Blomberg *et al.* 2003). The *K*-statistic was estimated using the ‘picante’ package in R program. We used the ‘Rtsne’ package in R to compute t-SNE dimensional reduction (Van der Maaten and Hinton 2008) and grouped traits and species to distinct clusters. The ‘Rtsne’ was run with ‘perplexity = 5’. A principal component analysis (PCA) was performed with the ‘prcomp’ function of the ‘vegan’ package in R program to analyse the associations among the traits. Multidimensional scaling (MDS) was conducted in SPSS 16.0 (SPSS Inc., Chicago, IL, USA) and was also used to verify the relationships of the traits.

## Results

### Variations in anatomical traits among species

In total, 22 root traits and 14 leaf traits of 21 *Dendrobium* species were studied. Coefficient of variation (CV) defined as the ratio of the standard deviation to the mean was used to measure trait variability. Leaf traits varied more than root traits for all *Dendrobium* species (Table 1). Leaf dry mass (*M*_L(D)_, CV = 145 %) and fresh mass (*M*_L(F)_, CV = 117 %) had the largest variation, while leaf area (LA) also varied greatly (CV = 97 %). Leaf water content (WC) had the smallest variation (CV = 15 %). For root traits, the area of vessels in cross section showed the greatest variation (*A*_ves_, CV = 88 %), while the area of velamen in cross section (*A*_vel_) also varied greatly (CV = 75 %). The ratio of radius of vascular cylinder to root radius (*R*_vc_/*r*) showed the smallest variation (CV = 17 %).

**Table 1.** Variations in leaf and root traits of tested *Dendrobium* species. SD: standard deviation; CV: coefficient of variation (%).

Traits	Abbr.	Function	Unit	Range	Mean	SD	CV (%)
Leaf fresh mass	*M*_L(F)_	Growth performance	g	0.05–2.42	0.54	0.63	117.49
Leaf dry mass	*M*_L(D)_	Growth performance	g	0.0072–0.49	0.08	0.12	145.04
Water content	WC	Water status	%	52.28–98.13	82.31	12.17	14.79
Leaf area	LA	Water loss	cm^2^	2.02–40.96	12.32	11.90	96.58
Leaf mass per area	LMA	Water conservation	g m^−2^	18.50–139.18	57.34	27.16	47.36
Leaf density	LD	Water conservation	kg m^−3^	57.62–210.33	138.70	46.63	33.62
Vein density	VD	Water transport	mm mm^−2^	1.60–5.64	2.80	1.08	38.39
Leaf thickness	LT	Water conservation	µm	157.31–899.75	446.11	217.42	48.74
Upper epidermal thickness	UET	Water conservation	µm	19.26–70.93	38.32	11.24	29.32
Upper cuticle thickness	UCT	Water conservation	µm	2.36–18.10	7.26	3.31	45.64
Lower epidermal thickness	LET	Water conservation	µm	9.55–56.69	24.44	9.93	40.65
Lower cuticle thickness	LCT	Water conservation	µm	1.01–12.75	5.92	3.25	54.89
Stomatal density	SD	Water loss	No. per mm^2^	36.72–108.16	67.44	22.22	32.95
Stomatal area	SA	Water loss	µm^2^	261.9–1160.0	623.39	194.38	31.18
Layer of velamen	LV	Water conservation	No.	3–10	5.76	2.05	35.53
Velamen thickness in cross section	VT	Water conservation	µm^2^	87.22–589.31	305.32	140.96	46.17
Root radius in cross section	*r*	Water absorbability	µm	413.02–1550.99	843.18	280.79	33.30
Velamen thickness/radius	VT/*r*	Water conservation	%	18.59–46.96	34.83	7.06	20.26
Velamen area in cross section	*A*_vel_	Water conservation and storage	mm^2^	0.23–4.65	1.49	1.12	75.09
Unit velamen cell length	vcl	Water storage	µm	20.45–72.71	43.14	13.92	32.28
Unit velamen cell width	vcw	Water storage	µm	13.46–45.59	27.67	7.67	27.73
Velamen cell length/width	vcl/vcw	Water storage		0.85–2.49	1.58	0.41	25.86
Area of velamen cell	*A*_vc_	Water storage	µm^2^	769.3–4472.1	1849.88	984.59	53.22
Number of exodermis cell	*N*_exo_	Water transport	No.	70–196	115.76	31.28	27.02
Number of exodermis passage cell	*N*_exopc_	Water transport	No.	1–13	6.85	3.05	44.49
Ratio of passage cell to exodermis cell	exopc%	Water transport	%	1.28–11.25	6.31	2.92	46.27
Number of endodermis cell	*N*_en_	Water transport	No.	32–100	54.95	17.94	32.65
Number of endodermis passage cell	*N*_enpc_	Water transport	No.	3.33–14	8.8	2.77	31.52
Ratio of passage cell to endodermis cell	enpc%	Water transport	%	4.76–20.00	16.6	3.96	23.88
Number of vessel	*N*_ves_	Water transport	No.	7–20	11.71	3.95	33.73
Diameter of vessel	*D*_ves_	Water transport	µm	13.74–65.43	27.62	10.61	38.40
Area of vessel in cross section	*A*_ves_	Water transport	µm^2^	152.12–2782.72	614.09	538.15	87.63
Root cortex thickness	RCT	Water storage	µm	157.25–624.20	291.48	104.54	35.87
Root cortex thickness/radius	RCT/*r*	Water storage	%	21.53–44.26	35.10	6.09	17.35
Radius of vascular cylinder	*R*_vc_	Water transport	µm	121.37–462.87	246.96	85.52	34.63
Radius of vascular cylinder/radius	*R*_vc_/*r*	Water transport	%	21.22–41.04	29.61	4.98	16.83
Elevation	EL		m	700–2500			29.74

In terms of the function that the leaf and root traits reflected, the traits related to water conservation showed relatively large variation. Among leaf traits, the CV values for leaf mass per area (LMA), leaf thickness (LT), upper cuticle thickness (UCT), lower cuticle thickness (LCT) were 47 %, 49 %, 46 % and 55 %, respectively. Among root traits, the CV values for velamen thickness (VT) and *A*_vel_ were 46 % and 75 %, respectively.

### Correlations between leaf and root traits in *Dendrobium*

For leaf traits, significant positive correlations were observed between LMA and LT (*r* = 0.85), UCT and LCT (*r* = 0.72 and 0.79, respectively) and lower epidermal thickness (LET, *r* = 0.68) **[see****Supporting Information—Table S1****]**. Leaf thickness (LT) was positively correlated with *M*_L(D)_ (*r* = 0.56), LMA (*r* = 0.85), UCT and LCT (*r* = 0.62 and 0.73, respectively), UET and LET (*r* = 0.53 and 0.74, respectively). Stomatal density (SD) was positively correlated with leaf density and vein density (LD and VD, *r* = 0.56 and 0.45, respectively), but negatively correlated with LET (*r* = −0.52). The LET was also positively correlated with UET, UCT and stomatal area (SA, *r* = 0.86, 0.58 and 0.46, respectively).

Traits related to root velamen were strongly correlated with root radius (*r*), whether or not phylogenetic effects were considered **[see****Supporting Information—Table S2****]**. For instance, VT and *A*_vel_ were positively correlated with root radius (*r* = 0.96 and 0.99, respectively). Velamen thickness (VT) was also positively correlated with the number of exodermis cells (*N*_exo_, *r* = 0.69) and endodermis cells (*N*_en_, *r* = 0.67), and the number of vessels (*N*_ves_, *r* = 0.62). Meanwhile, VT was positively correlated with root cortex thickness (RCT) and radius of vascular cylinder (*R*_vc_, *r* = 0.78 and 0.83, respectively), but negatively correlated with *R*_vc_/*r* (*r* = −0.52). The *N*_ves_ was not only positively correlated with the variables associated with velamen including LV, VT and *A*_vel_ (*r* = 0.59, 0.62 and 0.67, respectively), but also positively correlated with *N*_exo_, *N*_en_ and *N*_enpc_ (*r* = 0.87, 0.98 and 0.48, respectively).

**Figure 1.**
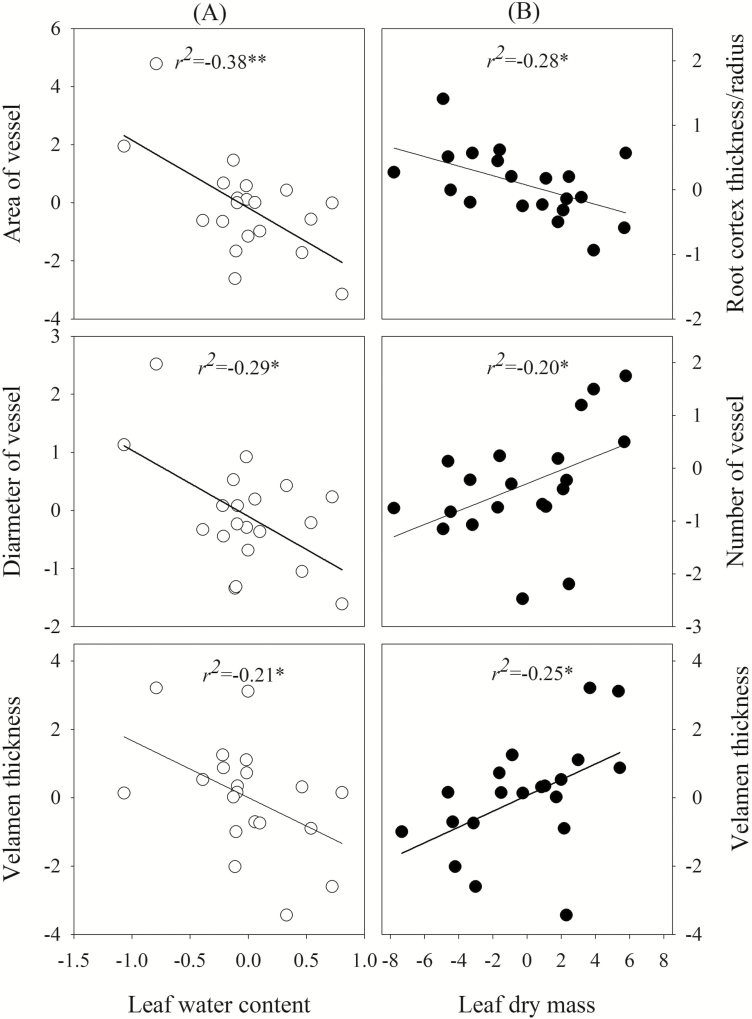
(A) Leaf water content was negatively correlated with root traits: cross-section area of vessel, diameter of vessel and velamen thickness (white circle); (B) leaf dry mass was positively correlated with root traits: velamen thickness and number of vessel, but negatively with the ratio of root cortex thickness to radius (black circle). Significance levels are expressed as follows: *P ≤ 0.05; **P ≤ 0.01. Data were corrected by PICs.

Several leaf and root traits were positively correlated (Table 2). *M*_L(D)_ was positively correlated with LV (*r* = 0.47), VT (*r* = 0.50), the ratio of velamen thickness to radius (VT/*r*, *r* = 0.58), *A*_vel_ (*r* = 0.45), *N*_en_ (*r* = 0.48) and *N*_ves_ (*r* = 0.45), but negatively correlated with the ratio of root cortex thickness to root radius (RCT/*r*, *r* = −0.53; Fig. 1). Leaf water content (WC) was negatively correlated with *A*_ves_, *D*_ves_ and VT (*r* = −0.62, −0.54 and −0.46, respectively; Fig. 1). Leaf area (LA) was positively correlated with VT/*r* (*r* = 0.47) and negatively correlated with RCT/*r* (*r* = −0.55). Leaf density (LD) was positively correlated with *N*_en_ and *N*_ves_ (*r* = 0.45 and 0.49, respectively). There were also positive correlations between LCT and VT (*r* = 0.53), VT/*r* (*r* = 0.46), *A*_vel_ (*r* = 0.53) and root radius (*r* = 0.51).

**Table 2.** Pearson’s correlation coefficients among leaf and root traits across 21 *Dendrobium* species. Data were corrected by PICs. Significant correlations are showed in boldface. See Table 1 for definitions of abbreviations. Asterisks denote significant levels: **P ≤ 0.01; *P ≤ 0.05.

Variables	LV	VT	*r*	VT/*r*	*A*_vel_	vcl	vcw	*A*_vc_	*N*_exo_	*N*_exopc_	*N*_en_	*N*_enpc_	*N*_ves_	*D*_ves_	*A*_ves_	RCT	RCT/*r*	*R*_vc_	*R*_vc_/*r*
*M*_L(F)_	0.21	0.11	0.02	0.24	0.07	−0.12	−0.23	−0.08	0.19	0.03	0.30	0.11	0.30	0.05	0.03	−0.11	−0.29	0.11	0.20
*M*_L(D)_	0.47*	0.50*	0.39	0.58**	0.45*	0.21	0.14	0.29	0.40	0.10	0.48*	0.15	0.45*	0.38	0.41	0.15	−0.53*	0.44	0.02
WC	−0.21	−0.46*	−0.41	−0.44	−0.44	−0.47*	−0.38	−0.41	−0.10	−0.18	−0.08	−0.07	−0.02	−0.54*	−0.62**	−0.25	0.37	−0.36	0.21
LA	0.31	0.31	0.18	0.47*	0.24	0.10	−0.06	0.08	0.20	0.04	0.31	0.03	0.27	0.35	0.38	−0.07	−0.55*	0.27	0.17
LMA	0.51*	0.59**	0.57**	0.48*	0.58**	0.31	0.42	0.50*	0.54*	0.15	0.54*	0.28	0.54*	0.25	0.27	0.47*	−0.23	0.51*	−0.26
LD	0.30	0.31	0.34	0.17	0.33	0.00	0.16	0.15	0.37	0.02	0.45*	0.20	0.49*	−0.04	0.04	0.26	−0.18	0.41	0.08
VD	−0.15	0.01	0.09	−0.14	0.05	0.26	0.40	0.32	−0.08	−0.08	−0.16	0.12	−0.16	0.14	0.12	0.13	0.09	0.03	−0.15
LT	0.31	0.38	0.34	0.35	0.37	0.28	0.30	0.38	0.30	0.12	0.26	0.16	0.24	0.25	0.23	0.29	−0.12	0.25	−0.28
UET	0.20	0.14	0.06	0.24	0.10	0.20	0.22	0.14	0.10	0.05	−0.06	−0.19	−0.02	0.27	0.15	−0.07	−0.28	−0.03	−0.23
UCT	0.30	0.43	0.43	0.33	0.43	0.25	0.48*	0.32	0.29	0.29	0.24	0.13	0.26	0.25	0.24	0.36	−0.16	0.34	−0.31
LET	0.41	0.35	0.26	0.44	0.31	0.29	0.25	0.26	0.29	0.20	0.16	0.03	0.18	0.31	0.20	0.12	−0.31	0.16	−0.29
LCT	0.42	0.53*	0.51*	0.46*	0.53*	0.32	0.46*	0.39	0.43	0.29	0.35	0.11	0.35	0.41	0.36	0.35	−0.35	0.43	−0.30
SD	−0.04	0.22	0.32	−0.02	0.27	0.15	0.22	0.31	0.21	0.03	0.31	0.24	0.34	−0.20	−0.10	0.39	0.13	0.31	−0.11
SA	0.24	0.03	0.01	0.06	0.02	−0.10	0.18	−0.12	0.11	0.18	−0.03	0.08	0.00	0.14	−0.04	−0.01	−0.06	−0.01	−0.06
EL	0.24	0.25	0.30	0.08	0.28	−0.07	−0.24	0.11	0.44	0.08	0.47*	0.31	0.57**	−0.14	−0.13	0.25	−0.12	0.30	−0.08

**Figure 2.**
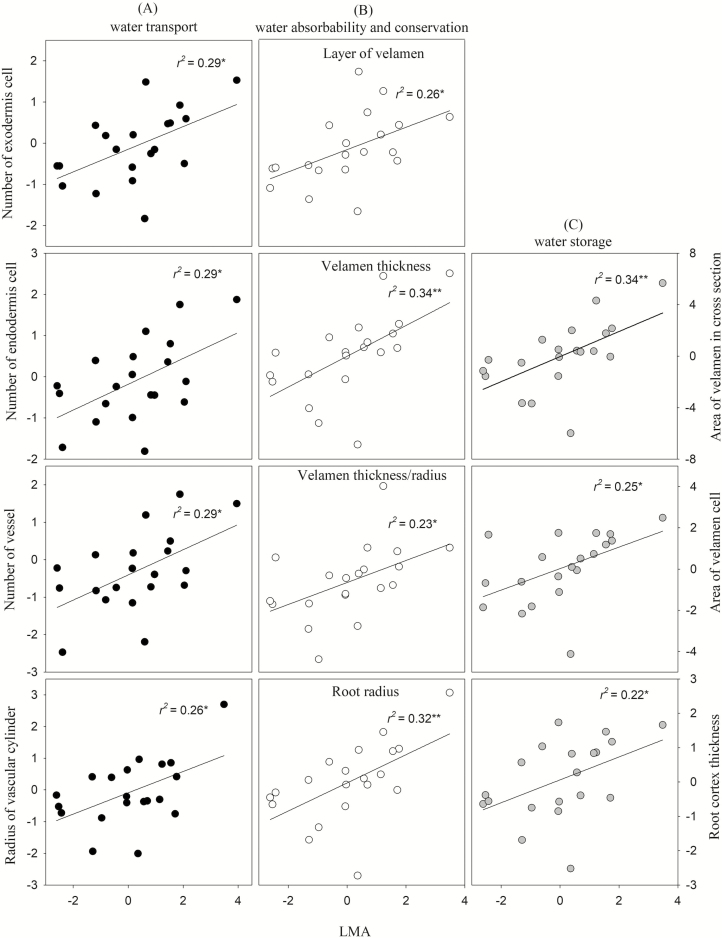
(A) Correlations between leaf mass per area (LMA) and root traits related to water transport (black circle), (B) water absorption and conservation (white circle), (C) water storage (grey circle). Significance levels are expressed as follows: *P ≤ 0.05; **P ≤ 0.01. Data were corrected by PICs.

Interestingly, LMA was positively correlated with root traits related to water absorbability (root radius, *r* = 0.57), water storage (*A*_vel_, *A*_vc_ and RCT, *r* = 0.58, 0.50 and 0.47, respectively), water transport (*N*_exo_, *N*_en_, *N*_ves_, *R*_vc_, *r* = 0.54, 0.54, 0.54 and 0.51, respectively) and water conservation (LV, VT, VT/*r*, *r* = 0.51, 0.59 and 0.48, respectively; Fig. 2).

### Influence of phylogeny and elevation on leaf and root traits in *Dendrobium*

To test whether variations observed in leaf and root traits were shaped by phylogeny or environmental factors, we tested traits in 21 *Dendrobium* species for phylogenetic signals using the *K*-statistic (Table 3). Almost all the traits showed a weak phylogenetic signal, except UET. This finding indicated that the effect of ecological variation on these traits overshadowed evolutionary constraints, especially LD (*K* = 0.721, *P* = 0.004), LV (*K* = 0.805, *P* = 0.035) and *R*_vc_/*r* (*K* = 0.606, *P* = 0.041).

**Table 3.** Phylogenetic signals of leaf and root traits in 21 *Dendrobium* species. Significant correlations are shown in boldface. Asterisks denote significant levels: **P ≤ 0.01; *P ≤ 0.05, respectively.

Trait	Phylogenetic signal	
	*K*	*P*
Leaf fresh mass (*M*_L(F)_)	0.425	0.664
Leaf dry mass (*M*_L(D)_)	0.439	0.416
Water content (WC)	0.584	0.082
Leaf area (LA)	0.487	0.230
Leaf mass per area (LMA)	0.402	0.723
Leaf density (LD)	**0.721**	0.004**
Vein density (VD)	0.385	0.742
Leaf thickness (LT)	0.366	0.854
Upper epidermal thickness (UET)	1.223	0.253
Upper cuticle thickness (UCT)	0.64	0.749
Lower epidermal thickness (LET)	0.861	0.531
Lower cuticle thickness (LCT)	0.554	0.803
Stomatal density (SD)	0.723	0.475
Stomatal area (SA)	0.92	0.363
Layer of velamen (LV)	**0.805**	0.035*
Velamen thickness (VT)	0.48	0.558
Root radius (*r*)	0.392	0.889
Velamen thickness/radius (VT/*r*)	0.639	0.116
Velamen area in cross section (*A*_vel_)	0.433	0.755
Unit velamen cell length (vcl)	0.6	0.321
Unit velamen cell width (vcw)	0.594	0.046
Velamen cell length/width (vcl/vcw)	0.579	0.343
Area of velamen cell (*A*_vc_)	0.382	0.828
Number of exodermis cell (*N*_exo_)	0.446	0.605
Number of exodermis passage cell (*N*_exopc_)	0.361	0.831
Ratio of passage cell to exodermis cell (exopc%)	0.367	0.809
Number of endodermis cell (*N*_en_)	0.547	0.494
Number of endodermis passage cell (*N*_enpc_)	0.37	0.802
Passage cell/endodermis cell (enpc%)	0.404	0.769
Number of vessel (*N*_ves_)	0.454	0.843
Diameter of vessel (*D*_ves_)	0.527	0.244
Area of vessel in cross section (*A*_ves_)	0.56	0.145
Root cortex thickness (RCT)	0.338	0.913
Root cortex thickness/radius (RCT/*r*)	0.641	0.162
Radius of vascular cylinder (*R*_vc_)	0.533	0.414
Vascular cylinder radius/radius (*R*_vc_/*r*)	**0.606**	0.041*

**Figure 3.**
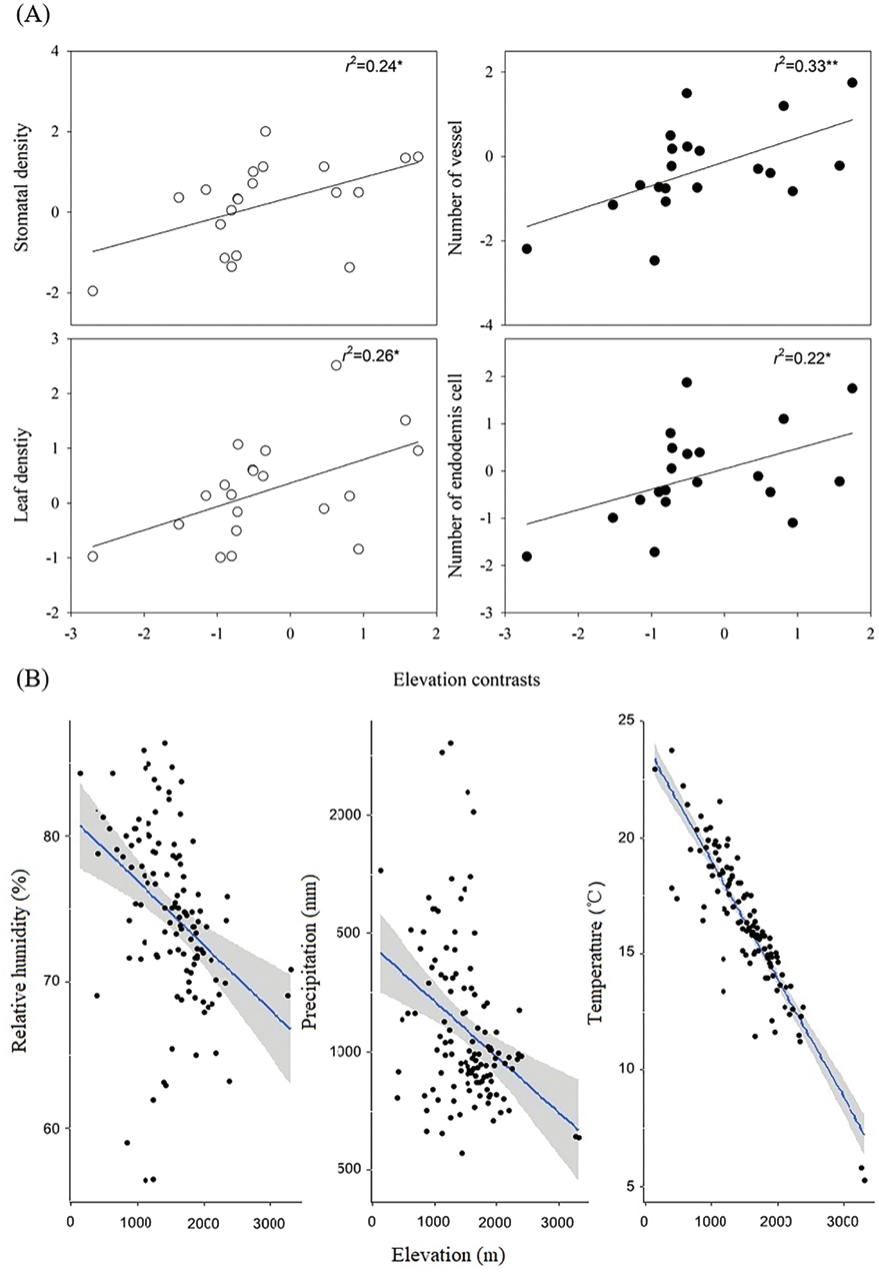
Elevation is positively correlated with (A) root traits (black circle): number of vessel and endodermis cell, and leaf traits (white circle): leaf density and stomatal density. Significance levels are expressed as follows: *P ≤ 0.05; **P ≤ 0.01. Data were corrected by PICs. (B) Variations of relative humidity, precipitation and temperature with elevation in Yunnan Province. Each scatterplot represents a meteorological station (*n* = 119).

We found that leaf traits such as LD and SD, root traits such as *N*_en_ and *N*_ves_ were positively correlated with elevation (Fig. 3A). In Yunnan Province, increase in elevation is accompanied by decreasing temperature, relative humidity and precipitation (Fig. 3B). These findings indicated that leaf and root traits in *Dendrobium* were affected by temperature and moisture level.

**Figure 4.**
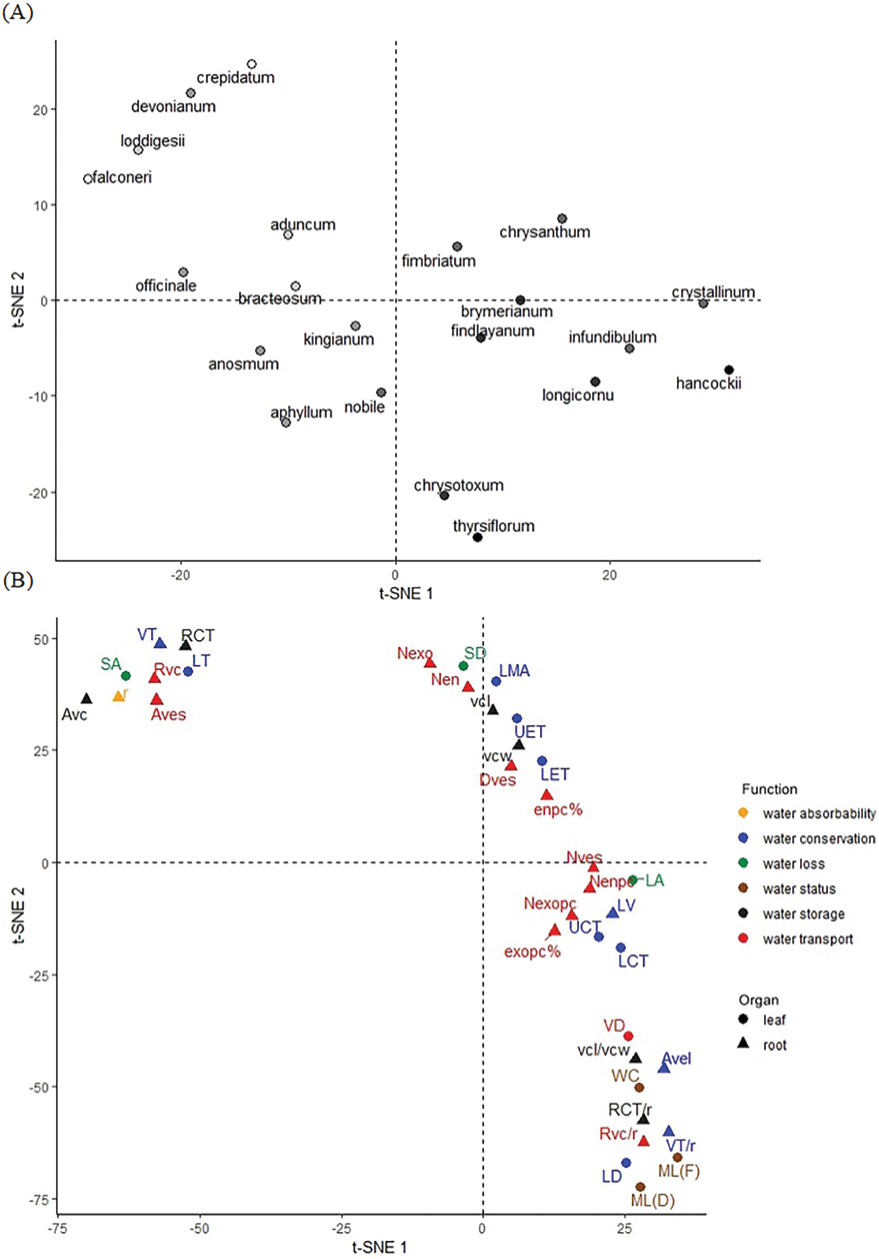
t-SNE (t-distributed stochastic neighbourhood embedding) visualization to compare the species and traits of *Dendrobium*. (A) Species were separated along zero axis of t-SNE 1. Dot colour was used to denote the relative size of the species root radius, and darker colours were used to denote thicker roots. (B) Traits were gathered in several parts with different function and belonging to different organs. Each dot denotes a trait. Colours denote corresponding function. The circle and triangle represent leaf and root traits, respectively.

The analysis based on the t-distributed stochastic neighbourhood embedding (t-SNE) showed the clustering results of the species and traits among *Dendrobium* (Fig. 4). The species were separated by the zero axis vertical to t-SNE 1. One group of species was those with thick roots and the other was those with thin roots (Fig. 4A). The leaf and root traits were gathered into four parts with different functions, and both leaf and root traits were included in each part (Fig. 4B). We also used the PCA and MDS to compare the leaf and root traits among *Dendrobium* species **[see****Supporting Information—Fig. S2****]**, and obtained results consistent with the t-SNE. This indicated that the functional traits tended to coupling between leaves and roots.

## Discussion

### Coordinated evolution of leaf and root traits within *Dendrobium*

**Figure 5.**
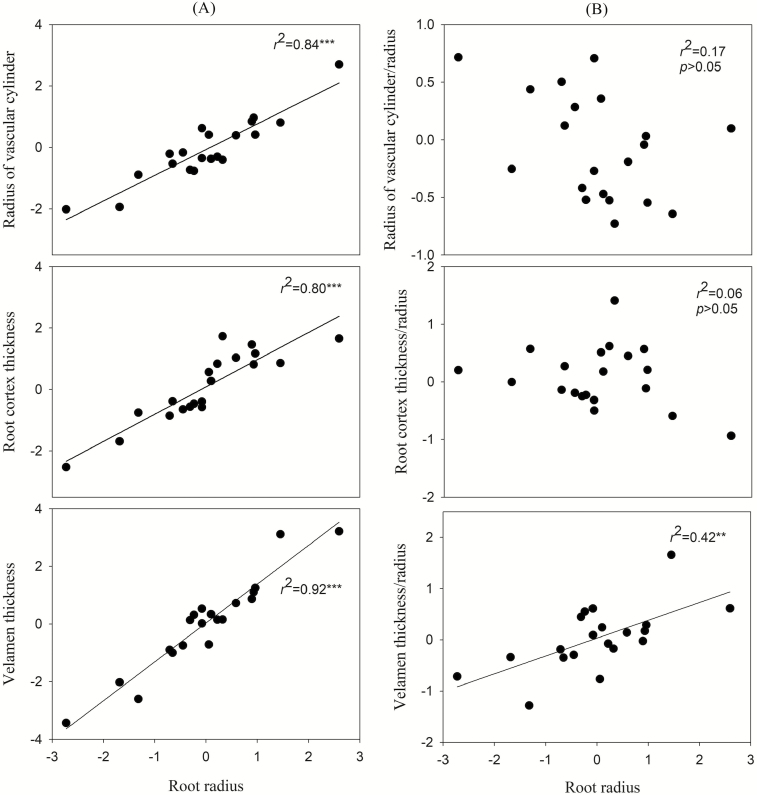
Correlations of root radius with velamen thickness, root cortex thickness and radius of vascular cylinder (A), and with the ratio of velamen thickness to radius, ratio of root cortex thickness to radius and ratio of vascular cylinder radius to root radius (B). Significance levels are expressed as follows: ***P ≤ 0.001; **P ≤ 0.01. Data were corrected by PICs.

Our study suggests that leaf and root traits in *Dendrobium* have evolved in coordination to cope with water stress, which is consistent with our hypothesis. Roots are the major organ for absorbing water and nutrients (Pregitzer *et al.* 2002; Guo *et al.* 2008; Liese *et al.* 2017). Most researches have focused on ‘fine roots’, which are defined as those <2 mm in diameter (Mommer and Weemstra 2012; Kong *et al.* 2014). For absorptive roots, the radius is a key trait because thicker roots have greater dependence on mycorrhizal fungi and may lead to a different absorptive strategy compared to thinner roots (Guo *et al.* 2008; Kong *et al.* 2015; Ma *et al.* 2018). In our study, the radius of the thickest root (1551 µm) was nearly 4-fold greater than the thinnest root (413 µm). Even the thinnest root exceeds the standard for thick roots (diameter > 470 µm) in a previous study (Kong *et al.* 2014). This indicates that the root traits of *Dendrobium* in this study may be different to the thin root traits of other plants. Meanwhile, root radius had significant positive relationships with velamen thickness, root cortex thickness and radius of vascular cylinder (Fig. 5A). This finding indicates that the variation in root radius may arise from the combined thickening of the velamen, cortex and vascular cylinder. We also found that the ratio of velamen thickness to root radius (VT/*r*) was positively correlated with root radius, but there were no relationships between root radius and the ratio of root cortex thickness to root radius (RCT/*r*), and the ratio of radius of vascular cylinder to root radius (*R*_vc_/*r*) (Fig. 5B). This suggests that the thicker roots of *Dendrobium* may be caused by a higher proportion of velamen. Thus, velamen thickness was a proxy for the root radius. This result conflicts with some previous researches on tree species that find a negative relationship between root density and root diameter, because the lower density of thick roots is caused by a larger proportion of root cortex (Chen *et al.* 2013; Kong *et al.* 2015). This may be because the roots of *Dendrobium* plants are exposed to the atmosphere. Velamen plays a crucial role in arboreal habitats (Joca *et al.* 2017). Although thicker velamen (due to greater numbers of cell layers) incurs greater construction costs (Enquist *et al.* 1999), it confers greater resistance to water loss and mechanical damage (Zotz and Hietz 2001). Thus, velamen is an important regulator to enhance the adaptability of *Dendrobium* plants to the environment.

Species with thicker root velamen had a higher leaf mass per area (LMA) and thicker leaf lower cuticle thickness (LCT). Meanwhile, in the species with higher leaf water content, the velamen thickness and area tended to be thinner in roots. Leaf dry mass is commonly used to measure the leaf strength and durability (Portillo-Estrada *et al.* 2015). The leaves with higher dry mass always have thicker laminas and higher tissue density because of their greater concentration of fibres and cell walls (Shipley and Vu 2002). Leaf mass per area (LMA) is used as an indicator of water and nutrient retention in plants (Witkowski and Lamont 1991), and a higher LMA represents a more conservative strategy. Greater LMA also brings a greater cost to the plant (Westoby *et al.* 2002). The average LMA is well known to be higher in low rainfall environments, owing to thicker leaves, denser tissue or both (Cunningham *et al.* 1999; Niinemets 2001). Likewise, leaf lower cuticle thickness is also related to water conservation (Zhang *et al.* 2012; Sun *et al.* 2014). All of the leaf traits mentioned above were correlated with root traits. This greatly supported our hypothesis that leaf and root traits were coordinated in terms of water conservation.

Leaf area was positively correlated with the ratio of velamen thickness to root radius (VT/*r*), but negatively correlated with the ratio of root cortex thickness to root radius (RCT/*r*). Leaf surfaces are the primary border of energy and mass exchange. Some important processes such as evapotranspiration and photosynthesis are directly proportional to leaf area (Agrawal *et al.* 2009). Previous studies have shown that lower leaf area helps plants prevent water loss in xeric conditions (Qin *et al.* 2019). The roots with larger proportions of velamen have a higher capacity for water conservation, but a larger leaf area means greater water loss. This may be because species with a conservative water use strategy tends to generate a larger total leaf area to offset the costs of construction in water conservation tissue (Reich *et al.* 1992; Westoby *et al.* 2002).

The results of the t-SNE showed that leaf and root traits were gathered, and not separated by functional category (Fig. 4B). This result is consistent with PCA and MDS. This provided further evidence that leaf and root traits coordinate to improve water utilization. Improvement of water utilization depends on the coupling of functional trait categories, supporting the idea of a whole-plant-based strategy. We also found the species were separated into two axes (Fig. 4A). This suggests that root radius may have an effect in driving leaf and root trait spectra, which is consistent with the findings in a previous study (Kong *et al.* 2015).

### The environment drives variation in water conservation traits within *Dendrobium*

Most leaf and root traits, especially the number of velamen layers, leaf density and the ratio of vascular cylinder radius to root radius, varied in response to the environments. This variation helps *Dendrobium* plants adapt to water stress. Two pieces of evidence support this finding: the patterns of leaf and root trait variations were consistent with the responses to environmental conditions in the arboreal habitats of *Dendrobium* (as discussed above), and leaf and root traits were correlated with elevational distribution.

No strong phylogenetic signal was detected in all leaf and root traits. This indicated that the effect of ecological factors on these traits overshadowed evolutionary constraints. Leaf density, layer of velamen and the ratio of vascular cylinder radius to root radius showed high adaptability to the environments (Table 3). This suggested that the environment, not phylogeny, was the main driver of leaf and root traits variation in *Dendrobium*. Leaf density responds generally to the changes in moisture (Xu and Zhou 2008). High leaf density can help plants cope with water stress (Witkowski and Lamont 1991). The increase in velamen layer numbers confers greater resistance to water loss and mechanical damage (Zotz and Hietz 2001). The vascular cylinder is responsible for the transport of water and nutrients to the shoot (Mellor *et al.* 2016). Ribeiro *et al.* (2019) reported that the increase in vascular cylinder diameter of *Glycine max* seedlings alleviates the effect aroused by water deficits. All these traits showed a strong relationship with environmental factors, and indicated that *Dendrobium* have a great capacity to withstand drought stress. But somewhat contradictory to our result, a study on leaf functional traits in *Dendrobium* found that phylogeny has a significant effect on leaf density and leaf upper cuticle thickness, although most traits measured also have weak signals (Sun *et al.* 2014). The discrepancy was probably caused by different materials, a wider diversity of species and cultivation conditions than in our study.

We found that elevational distribution was positively correlated with root traits such as the number of endodermis cell and vessel, and with leaf traits such as leaf density and stomatal density. All these traits are related to water use efficiency. The endodermis not only separates the vascular cylinder and provides a diffusion barrier (Roppolo *et al.* 2011), but also functions as a protective layer during drought (Ranathunge *et al.* 2003). When plants are deprived of water, the endodermis resists water movement from the stele to the outside, allowing internal layers to survive (Stasovski and Peterson 2011). A previous study has shown that water transport efficiency is promoted by increased number of vessels with a larger diameter (Dickison 2000). In contrast, drought can lead to a higher proportion of narrower (less efficient) vessels and decreased vessel numbers (Durante *et al.* 2011; Jupa *et al.* 2016). Moreover, some studies have shown that a water deficit leads to an increase in stomatal density, which is positively correlated with water use efficiency (Martínez *et al.* 2007; Xu and Zhou 2008).

Taken together, the significant correlations between elevation with endodermis and vessels number, leaf density and stomatal density indicated that a higher elevation tended to select traits that increased water use efficiency in *Dendrobium*. In Yunnan Province, high elevation is often accompanied by lower temperature and humidity (Fig. 3B). The number of epiphytic orchid species decreases with increasing elevation (Zhang *et al.* 2015). This indicates that a low moisture level is an important factor limiting the distribution of epiphytic orchids in high-altitude areas. We speculated that the species with thicker velamen may be more adapted to higher elevations as the velamen has the function of retaining moisture and warmth in roots. Although endodermis and vessels number were positively correlated with velamen thickness, elevation was not correlated with velamen thickness. It would be helpful to investigate the role of temperature and moisture levels in measuring the capacity for plants to adapt to the potential changing environmental conditions in the future.

## Conclusions

We proposed a model of interaction between leaf and root traits of *Dendrobium* which is an important epiphytic taxon. The majority of leaf and root traits were shaped by the environment rather than evolutionary constraints. To maintain water balance and improve water use efficiency, leaf and root traits showed close coordination in *Dendrobium*. The traits related to water uptake and conservation might play an important role in helping *Dendrobium* species to adapt to cold and dry conditions at high elevations. The results of this study confirmed the plant economic hypothesis, which states that plant populations adapt to the environment through coordinated leaf and root trait evolution. These findings improve our understanding of the interactive pattern of leaf and root traits in epiphytes.

## Supporting Information

The following additional information is available in the online version of this article—

Figure S1. Phylogenetic relationships and ecological information across 21 *Dendrobium* species.

Figure S2. (A) Principal component analysis (PCA) and (B) multidimensional scaling (MDS) are used to compare leaf and root traits among 21 *Dendrobium* species.

Table S1. Coefficients of Pearson’s correlations and phylogenetically independent contrast correlations among leaf traits, and between leaf traits and elevation across 21 *Dendrobium* species.

Table S2. Coefficients of Pearson’s correlations and phylogenetically independent contrast correlations among root traits across 21 *Dendrobium* species.

plaa034_suppl_Supplementary_Material

## Data Availability

All data used in this study are available at https://osf.io/8dkur.
