# Supplementary material for: Correlated evolution of leaf and root anatomic traits in Dendrobium (Orchidaceae)
Source: AoB Plants. 2020 Jul 20;12(4):plaa034. doi: 10.1093/aobpla/plaa034 (PMC7426005; doi:10.1093/aobpla/plaa034)
Supplement: plaa034_suppl_Supplementary_Material [file plaa034_suppl_supplementary_material.docx]

**Table S1** Coefficients of Pearson’s correlations and phylogenetically independent contrast correlations among leaf traits, and between leaf traits and elevation across 21 *Dendrobium* species.

|  | M_L(F)_ | M_L(D)_ | WC | LA | LMA | LD | VD | LT | UET | UCT | LET | LCT | SD | SA | EL |
| --- | --- | --- | --- | --- | --- | --- | --- | --- | --- | --- | --- | --- | --- | --- | --- |
| M_L(F)_ |  | 0.8*** | 0.37 | 0.81*** | 0.38 | -0.04 | -0.62** | 0.37 | -0.10 | -0.21 | 0.27 | 0.02 | -0.35 | 0.04 | 0.02 |
| M_L(D)_ | 0.73*** |  | -0.16 | 0.9*** | 0.68*** | 0.12 | -0.51* | 0.56** | 0.22 | 0.19 | 0.54* | 0.44* | -0.27 | 0.03 | 0.06 |
| WC | 0.33 | -0.30 |  | -0.20 | 0.00 | -0.12 | -0.3 | 0.06 | -0.25 | -0.23 | -0.12 | -0.31 | -0.12 | 0.05 | -0.19 |
| LA | 0.73*** | 0.91*** | -0.38 |  | 0.30 | 0.08 | -0.49* | 0.23 | 0.03 | -0.18 | 0.30 | 0.11 | -0.35 | 0.00 | 0.11 |
| LMA | 0.35 | 0.65** | 0.01 | 0.28 |  | 0.13 | -0.29 | 0.85*** | 0.43 | 0.72*** | 0.68*** | 0.79*** | 0.00 | 0.06 | -0.05 |
| LD | 0.04 | 0.40 | -0.33 | 0.33 | 0.34 |  | 0.08 | -0.40 | -0.24 | 0.10 | -0.22 | 0.00 | 0.56* | 0.14 | 0.51* |
| VD | -0.51* | -0.38 | -0.17 | -0.38 | -0.19 | 0.13 |  | -0.31 | 0.06 | 0.01 | -0.23 | -0.09 | 0.45* | -0.02 | 0.00 |
| LT | 0.30 | 0.31 | 0.26 | 0.02 | 0.69*** | -0.45* | -0.28 |  | 0.53* | 0.62** | 0.74*** | 0.73*** | -0.29 | -0.02 | -0.31 |
| UET | -0.23 | 0.11 | -0.20 | -0.06 | 0.37 | -0.08 | 0.14 | 0.41 |  | 0.59** | 0.86*** | 0.74*** | -0.44 | 0.36 | -0.22 |
| UCT | -0.33 | 0.11 | -0.23 | -0.19 | 0.61** | 0.11 | 0.08 | 0.49* | 0.66** |  | 0.58** | 0.9*** | 0.10 | 0.24 | -0.16 |
| LET | 0.12 | 0.40 | -0.11 | 0.18 | 0.63** | -0.04 | -0.16 | 0.63** | 0.86*** | 0.61** |  | 0.81*** | -0.52* | 0.46* | -0.24 |
| LCT | -0.12 | 0.35 | -0.33 | 0.07 | 0.71*** | 0.13 | -0.03 | 0.57** | 0.75*** | 0.89*** | 0.81*** |  | -0.08 | 0.31 | -0.10 |
| SD | -0.26 | -0.19 | -0.04 | -0.27 | 0.05 | 0.40 | 0.43 | -0.26 | -0.43 | 0.04 | -0.52* | -0.10 |  | -0.30 | 0.49* |
| SA | -0.02 | 0.10 | -0.11 | 0.05 | 0.14 | 0.24 | 0.01 | -0.05 | 0.40 | 0.30 | 0.53* | 0.40 | -0.34 |  | -0.16 |
| EL | -0.01 | 0.08 | -0.28 | 0.17 | -0.14 | 0.20 | -0.17 | -0.29 | -0.15 | -0.10 | -0.22 | -0.05 | 0.27 | -0.10 |  |

Lower diagonal, Pearson’s correlation coefficients; Upper diagonal, correlation coefficients of phylogenetically independent contrast. Significant correlations are showed in boldface. Asterisks denote significant levels: ***, *P*≤0.001; **, *P*≤0.01; *, *P*≤0.05, respectively. See Table 1 for definitions of abbreviations.

**Table S2** Coefficients of Pearson’s correlations and phylogenetically independent contrast correlations among root traits across 21 *Dendrobium* species.

|  | LV | VT | r | VT/r | A_vel_ | vcl | vcw | vcl/vcw | A_vc_ | N_exo_ | N_exopc_ | exopc% | N_en_ | N_enpc_ | enpc% | N_ves_ | D_ves_ | A_ves_ | RCT | R_vc_ | RCT/r | Rvc/r |
| --- | --- | --- | --- | --- | --- | --- | --- | --- | --- | --- | --- | --- | --- | --- | --- | --- | --- | --- | --- | --- | --- | --- |
| LV |  | **0.81***** | **0.79***** | **0.66**** | **0.81***** | 0.36 | 0.35 | 0.13 | **0.63**** | **0.77***** | 0.02 | -0.32 | **0.62**** | **0.45*** | -0.08 | **0.59**** | 0.23 | 0.20 | **0.68**** | **0.71***** | -0.28 | -0.38 |
| VT | **0.79***** |  | **0.96***** | **0.84***** | **0.99***** | **0.72***** | **0.58**** | 0.37 | **0.86***** | **0.69**** | 0.17 | -0.15 | **0.67**** | 0.43 | -0.13 | **0.62**** | 0.28 | 0.34 | **0.78***** | **0.83***** | -0.39 | **-0.52*** |
| r | **0.79***** | **0.94***** |  | **0.65**** | **0.99***** | **0.63**** | **0.59**** | 0.25 | **0.83***** | **0.81***** | 0.13 | -0.24 | **0.77***** | **0.56**** | -0.09 | **0.71***** | 0.35 | 0.42 | **0.89***** | **0.92***** | -0.25 | -0.42 |
| VT/r | **0.61**** | **0.87***** | **0.65**** |  | **0.75***** | **0.71***** | 0.43 | **0.50*** | **0.70***** | 0.30 | 0.22 | 0.05 | 0.32 | 0.09 | -0.18 | 0.30 | 0.08 | 0.13 | 0.39 | **0.46*** | **-0.57**** | **-0.58**** |
| A_vel_ | **0.8***** | **0.99***** | **0.98***** | **0.77***** |  | **0.68**** | **0.59**** | 0.31 | **0.85***** | **0.76***** | 0.15 | -0.2 | **0.73***** | **0.51*** | -0.11 | 0.67** | 0.32 | 0.38 | **0.85***** | **0.88***** | -0.32 | **-0.47*** |
| vcl | 0.05 | **0.55*** | 0.43 | **0.6**** | **0.50*** |  | **0.65**** | **0.66**** | **0.81***** | 0.19 | 0.16 | 0.06 | 0.24 | 0.21 | 0.01 | 0.18 | 0.07 | 0.19 | **0.56**** | 0.43 | -0.16 | **-0.61**** |
| vcw | 0.15 | **0.48*** | **0.46*** | 0.40 | **0.48*** | **0.66**** |  | -0.14 | **0.7**** | 0.24 | 0.23 | -0.10 | 0.22 | 0.21 | 0.03 | 0.15 | 0.36 | 0.40 | 0.55* | **0.50*** | -0.1 | -0.35 |
| vcl/vcw | -0.10 | 0.19 | 0.06 | 0.34 | 0.13 | **0.58**** | -0.24 |  | 0.37 | 0.02 | 0.20 | 0.16 | 0.10 | 0.07 | -0.01 | 0.10 | -0.26 | -0.15 | 0.20 | 0.08 | -0.12 | **-0.45*** |
| A_vc_ | 0.43 | **0.76***** | **0.7***** | **0.68***** | **0.74***** | **0.77***** | **0.74***** | 0.19 |  | **0.50*** | -0.01 | -0.22 | 0.43 | 0.40 | 0.04 | 0.37 | 0.15 | 0.24 | **0.79***** | **0.61**** | -0.1 | **-0.69***** |
| N_exo_ | **0.77***** | **0.58**** | **0.75***** | 0.19 | **0.67***** | -0.11 | 0.01 | -0.15 | 0.24 |  | -0.11 | **-0.53*** | **0.9***** | 0.48 | -0.29 | **0.87***** | 0.34 | 0.34 | **0.72***** | **0.86***** | -0.22 | -0.06 |
| N_exopc_ | 0.05 | 0.18 | 0.15 | 0.19 | 0.17 | 0.12 | -0.09 | 0.25 | -0.05 | -0.08 |  | **0.9***** | -0.05 | **0.5*** | **0.57**** | 0.01 | 0.16 | 0.09 | 0.11 | 0.06 | -0.04 | -0.19 |
| exopc% | -0.31 | -0.11 | -0.22 | 0.07 | -0.17 | 0.15 | -0.08 | 0.29 | -0.16 | **-0.53*** | **0.89***** |  | -0.44 | 0.22 | **0.61**** | -0.36 | -0.01 | -0.07 | -0.21 | -0.33 | 0.06 | -0.14 |
| N_en_ | **0.63**** | **0.6**** | **0.73***** | 0.27 | **0.67***** | -0.03 | 0.08 | -0.12 | 0.19 | **0.85***** | -0.03 | -0.42 |  | **0.45*** | -0.40 | **0.98***** | 0.24 | 0.31 | **0.65**** | **0.88***** | -0.27 | 0.07 |
| N_enpc_ | **0.46*** | 0.32 | **0.51*** | -0.03 | 0.42 | -0.08 | 0.00 | -0.10 | 0.10 | **0.55**** | **0.45*** | 0.12 | **0.51*** |  | **0.64**** | 0.48* | 0.28 | 0.27 | **0.70***** | 0.53* | 0.29 | -0.21 |
| enpc% | -0.11 | -0.23 | -0.16 | -0.29 | -0.20 | -0.06 | -0.08 | 0.01 | -0.08 | -0.23 | **0.5*** | 0.53* | -0.42 | **0.57**** |  | -0.36 | 0.08 | 0.01 | 0.16 | -0.22 | **0.54*** | -0.28 |
| N_ves_ | **0.58**** | **0.51*** | **0.65**** | 0.20 | **0.59**** | -0.08 | 0.06 | -0.17 | 0.15 | **0.8***** | 0.06 | -0.32 | **0.95***** | **0.52*** | -0.36 |  | 0.13 | 0.19 | **0.61**** | **0.80***** | -0.24 | 0.03 |
| D_ves_ | 0.35 | 0.27 | 0.39 | 0.03 | 0.33 | -0.09 | 0.15 | -0.28 | -0.02 | 0.44* | 0.15 | -0.07 | 0.38 | 0.42 | 0.08 | 0.31 |  | **0.95***** | 0.18 | **0.52*** | -0.39 | 0.31 |
| A_ves_ | 0.28 | 0.31 | 0.41 | 0.08 | 0.36 | 0.02 | 0.21 | -0.20 | 0.06 | 0.38 | 0.10 | -0.09 | 0.41 | 0.35 | -0.02 | 0.33 | **0.95***** |  | 0.26 | **0.59**** | -0.35 | 0.3 |
| RCT | **0.6**** | **0.69***** | **0.84***** | 0.32 | **0.78***** | 0.38 | 0.41 | 0.05 | **0.66**** | **0.66**** | 0.11 | -0.21 | **0.53*** | **0.62**** | 0.15 | **0.48*** | 0.16 | 0.18 |  | **0.77***** | 0.21 | -0.5* |
| R_vc_ | **0.72***** | **0.75***** | **0.87***** | 0.40 | **0.82***** | 0.15 | 0.29 | -0.12 | 0.37 | **0.82***** | 0.09 | -0.31 | **0.89***** | **0.56**** | -0.26 | **0.79***** | **0.63**** | **0.65**** | **0.64**** |  | -0.35 | -0.02 |
| RCT/r | -0.38 | **-0.48*** | -0.33 | **-0.61**** | -0.41 | -0.11 | -0.12 | -0.01 | -0.11 | -0.19 | -0.07 | 0.03 | -0.37 | 0.18 | **0.54*** | -0.32 | -0.42 | -0.43 | 0.24 | **-0.45*** |  | -0.02 |
| R_vc_/r | -0.11 | -0.35 | -0.22 | **-0.47*** | -0.29 | **-0.55**** | -0.33 | -0.35 | **-0.65**** | 0.18 | -0.12 | -0.18 | 0.35 | 0.12 | -0.21 | 0.32 | **0.49*** | **0.49*** | -0.38 | 0.28 | -0.27 |  |

Lower diagonal, Pearson’s correlation coefficients; Upper diagonal, correlation coefficients of phylogenetically independent contrasts. Significant correlations are showed in boldface. Asterisks denote significant levels: ***, *P*≤0.001; **, *P*≤0.01; *, *P*≤0.05, respectively. See Table 1 for definitions of abbreviations.


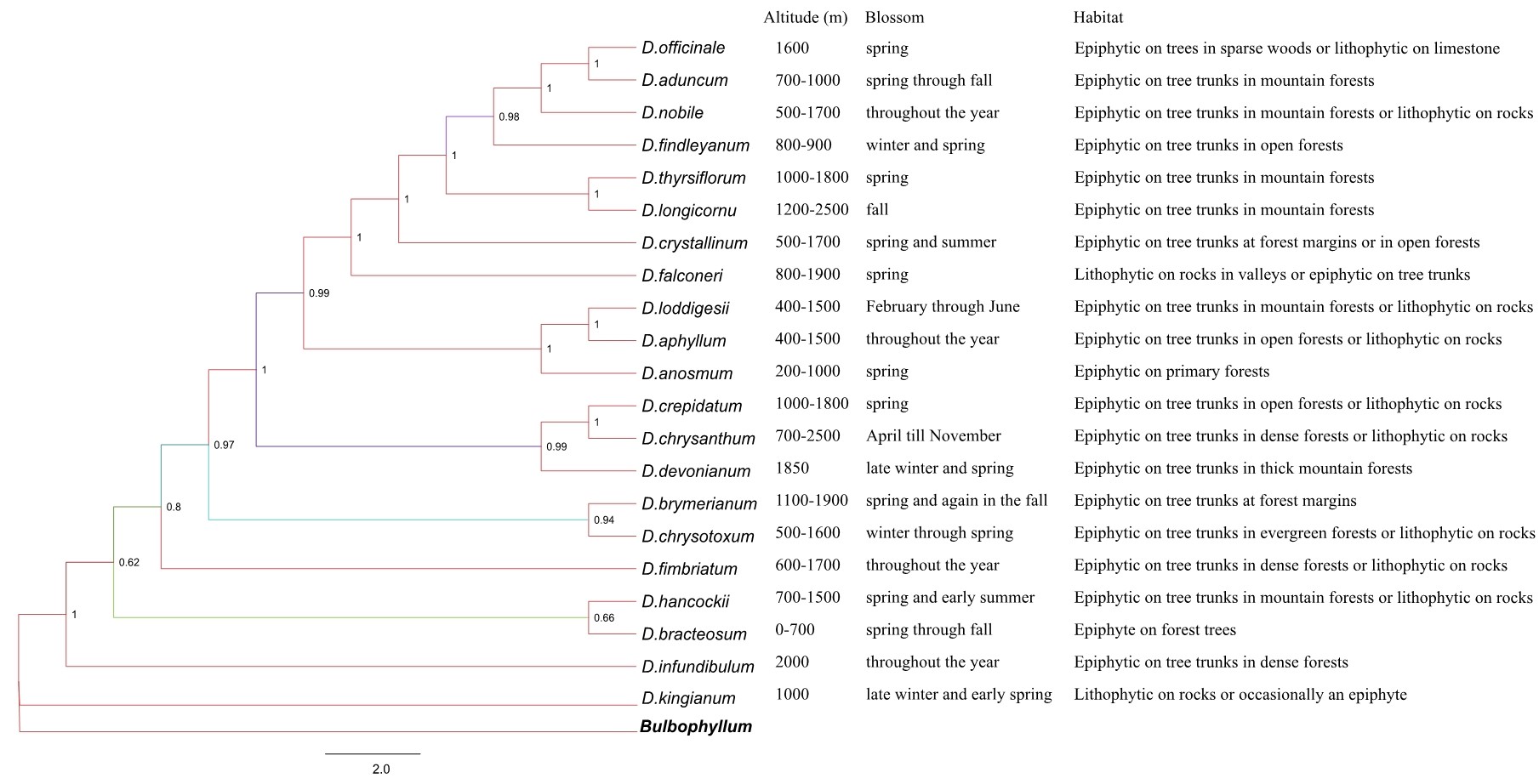


**Fig. S1** Phylogenetic relationships and ecological information across 21 *Dendrobium* species. Phylogram was generated by MrBayes, using concatenated dataset of ITS, *rbc*L, *mat*K-*trn*K, and *trn*H-*psb*A sequences download from GenBank. Numbers associated with nodes are maximum-likelihood bootstrap value.


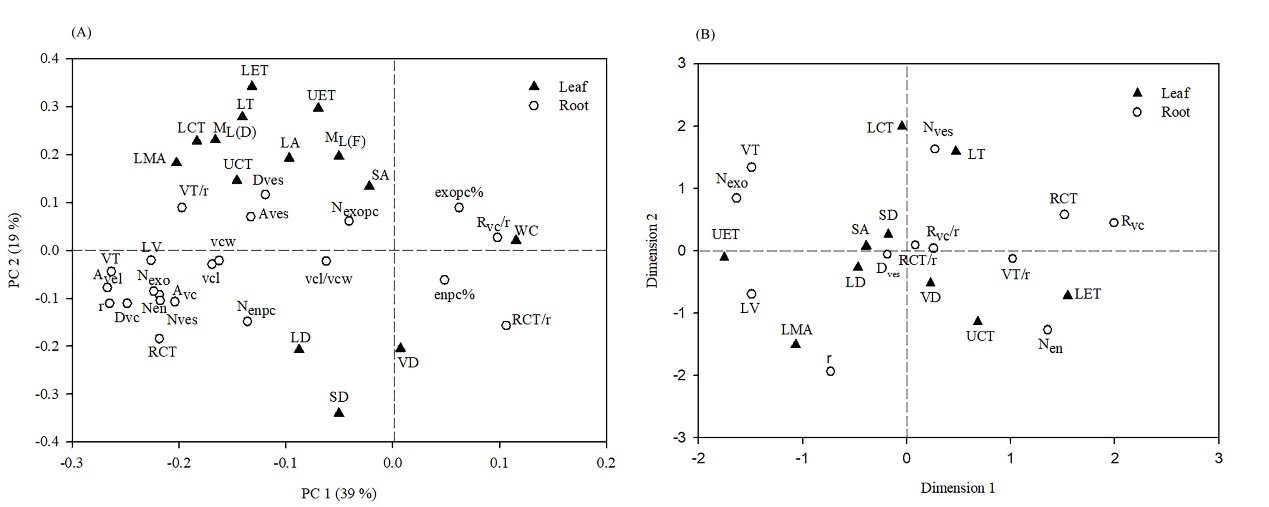
 **Fig.S2** **(A)** Principal component analysis (PCA) and **(B)** multidimensional scaling (MDS) are used to compare leaf and root traits among 21 *Dendrobium* species. The circles and triangles represent leaf and root traits, respectively.
